# Factors associated with late presentation of cervical cancer cases at a district hospital: a retrospective study

**DOI:** 10.1186/s12889-018-6065-6

**Published:** 2018-10-03

**Authors:** Priscilla Dunyo, Kofi Effah, Emilia Asuquo Udofia

**Affiliations:** 1 Department of Population, Family and Reproductive Health, School of Public Health, University of Ghana, Legon, Accra Ghana; 2 Obstetric and Gynecological Department/Cervical Cancer Screening and Training Center, Catholic Hospital, Battor, Ghana; 3 Department of Community Health, School of Public Health, University of Ghana, Legon, Accra Ghana

**Keywords:** Cervical cancer, Ghana, Oncology, Screening, Prevention

## Abstract

**Background:**

Cervical cancer is the leading and most common female cancer among women in Ghana. Although there are screening methods to detect premalignant lesions for treatment, screening coverage in Ghana is 2.8% and late presentation of cases complicates treatment efforts.

This study examined the sociodemographic, clinical and histological characteristics associated with late presentation of cervical cancer cases attending Gynecological Oncology care at Catholic Hospital, Battor.

**Methods:**

One hundred and fifty-seven medical records of confirmed cases of cervical cancer reporting to the Outpatient Obstetrics and Gynecology Department between 2012 and 2016 were reviewed. Relevant data were retrieved using abstraction forms. Socio demographic variables investigated were level of education attained, marital status, National Health Insurance Scheme membership, employment status, place of residence and distance from hospital. Clinical variables included intermenstrual/postmenopausal bleeding, previous screening history, previous smoking history, age at menarche and number of children. Histological variables included subtypes of tumour and characteristics of tumour. Pearson’s chi-square test and logistic regression analysis were used to determine correlates of late stage at presentation with cervical cancer. Sensitivity analysis was performed to assess the effect of missing data.

**Results:**

Approximately two-thirds (65.97%) of the cases presented in advanced stages of cervical cancer. Level of education, age at menarche and previous screening history were included in a regression model and adjusted for age. Age at menarche (*n* = 66) was eliminated from the model after sensitivity analysis. Among the remaining variables, only previous screening history was predictive of late stage at presentation of cervical cancer cases. Previously unscreened cases of cervical cancer were nearly four times more likely to present late, compared to those who had been screened previously (OR 3.91; 95% CI 1.43–10.69). No association was observed with sociodemographic and histological characteristics.

**Conclusion:**

Lack of previous screening was associated with late presentation of cervical cancer at Catholic Hospital, Battor. Efforts to promote early cervical cancer screening should be intensified and future studies may explore an association with age at menarche.

**Electronic supplementary material:**

The online version of this article (10.1186/s12889-018-6065-6) contains supplementary material, which is available to authorized users.

## Background

Cervical cancer has been reported to account for a third of all female cancers in Ghana and the World Health Organization (WHO) has predicted that it will account for 5000 new cases by the year 2025 [1]. It further predicts 3361 deaths will occur annually. In spite of the grim picture painted, there is no organized national screening programme in Ghana [2]. As a first step, a national policy was developed in 2011 as part of the National Cancer Control Plan. The policy recommends Visual Inspection with Acetic Acid (VIA) as a screening method, with cryotherapy as treatment, for women aged 25–45 years found to have a pre-malignant lesion. Cytology has been recommended for women aged 45 years and older [3].

Screening coverage for cervical cancer in Ghana is reportedly low at 2.8%, with the majority of women diagnosed when the disease is advanced [1]. This is not different from what occurs in other developing countries. For instance, in a hospital based, cross sectional study in Uganda, 66% of the study participants reported in advanced stages of cervical cancer [4]. Another study conducted in Tanzania reported that 63.9% of its study participants presented late to the healthcare facility [5]. An ecological study investigated population-level exposure to risk factors in relation to late stage cervical cancer and found that gross domestic product, HIV infection, non-use of condoms, high parity and lack of formal education were significant predictors [6]. A recent study conducted in two regions in Ghana, also reported that late stage presentation was common [7].

Previous studies have shown that low socioeconomic status is associated with late presentation of cervical cancer [8, 9]. In addition, other factors such as race, age at diagnosis [8, 10], insurance status [11, 12], marital status [4, 13], educational status [14], place of residence [15, 16], number of children [4] and screening practices [17] have been associated with the stage at presentation, which in turn affect survival rates. The ability to identify abnormal genital tract bleeding symptoms (post-menopausal bleeding or intermenstrual bleeding) can affect health seeking behaviour, while indicative results from previous screening, family history of cervical cancer, personal history of sexually transmitted infections and smoking contribute to the development of cervical cancer [13, 14]. There is also evidence that biological behaviour of the tumour predicts the stage at diagnosis [5, 18, 19].

Human papilloma virus DNA testing is the recommended method of primary screening for cervical cancer if all resources are available [20] The Papanicolau test (Pap smear) and VIA are other screening tests which have been used to detect premalignant lesions. Early detection of lesions with appropriate intervention reduces related morbidity and mortality. Countries that have established screening programs in the last few decades have seen a significant decline in incidence and deaths due to cervical cancer. Examples include Norway, Sweden, Finland [21, 22]. At the Catholic Hospital in Battor, available records indicate that nearly 50% of women seeking gynaecological oncology care, are women with cervical cancer. Most of these women present late and the factors associated with their late stage presentation are uncertain. The hospital provides screening services at low cost to patients. In spite of this, service utilization is low and raises concerns about barriers to service use. With the aim of improving uptake of screening services, a study was undertaken to identify sociodemographic, clinical and histological characteristics associated with late presentation of cervical cancer cases reporting to the hospital in the period 2012 to 2016.

## Method

### Study design

A cross sectional analytical study design where review of hospital records available at the Outpatient Gynaecological Oncology clinic was conducted. All records of cases seen at the clinic at the Catholic Hospital, Battor and diagnosed with cervical cancer between 2012 and 2016 were reviewed to identify relevant variables for the study.

### Patient record selection

**Fig. 1:**
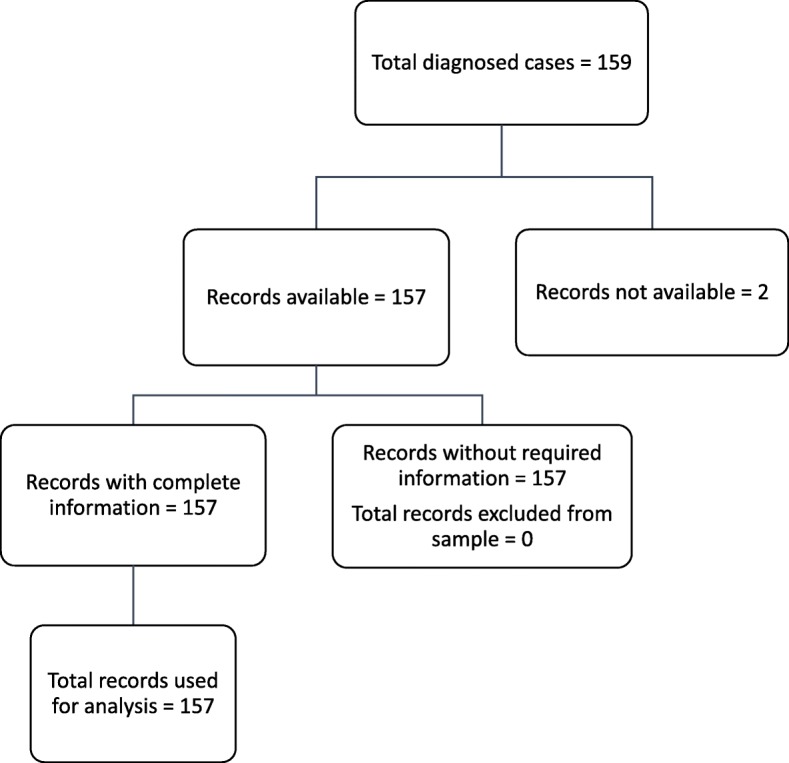
Schematic diagram showing the record selection process

The hospital filing system captured histological reports and medical records of the patients at the gynaecological unit. A total of 159 women diagnosed with cervical cancer were listed in the period under review (Fig. 1). All but two missing records, were collected, arranged chronologically by year of diagnosis and then by month. PD and a trained research assistant selected all records that had most of the variables investigated in the study.

### Data collection

**Fig. 2:**
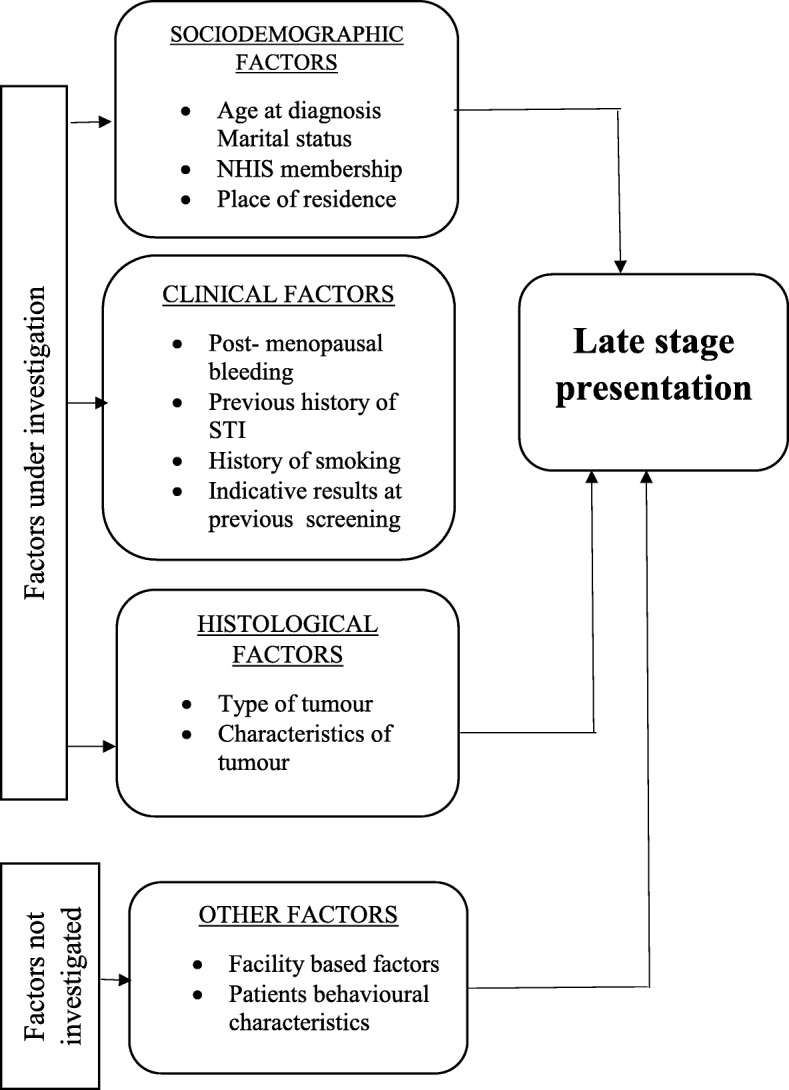
Conceptual framework for the study

Data abstraction from the patient records was done manually by a trained record clerk using abstraction forms consistent with literature [23]. The abstraction form had separate sections for sociodemographic variables (age, highest level of education attained, marital status, NHIS, employment status, place of residence and distance from hospital), clinical variables (intermenstrual/postmenopausal bleeding, previous screening history, previous smoking history, age at menarche, number of children), histological variables (subtypes of tumour and characteristics of tumour) and stage at presentation (early stage – Stages I & IIA; late stage – Stages IIB - IV). The selected characteristics were informed by reviewed literature and availability of data in patient records based on the framework in Fig. 2.

### Statistical analysis

Fifty randomly selected abstraction forms were re-checked by EK and PD by comparing with actual records to ensure entries were correct. All data were coded and doubly entered into electronic spreadsheets using Stata version 14. Each variable was scrutinized visually before running the analysis. Descriptive statistics generated included percentages and frequencies of the investigated variables. Some variables such as parity and age of onset of menarche were grouped into dichotomous variables. For parity, two categories (0–4 and 5–12) were used because the total fertility rate in Ghana, being the maximum number of children a woman is expected to have is 4.2 [24]. Additionally, the descriptive summaries for continuous variables yielded a median value of 5 and mean value of 4.85 for parity. This was applied as a threshold to divide parity into two categories namely < 5 and ≥ 5 children. Categories for age at menarche were determined in like manner. This variable had a median of 16 years, therefore two groups were created namely: 7–15 years and 16–25 years. Furthermore, a Turkish study indicated that the lowest prevalence of cervical cancer was found among women who reported menarche at the age of 15 years or older [25]. The authors were unable to find a similar local study reporting an association between age at menarche and cervical cancer. Cross tabulation of the investigated characteristics and the late stage at presentation of cervical cancer cases was performed using the Pearson’s chi-square test and Fischer’s exact test where appropriate. Variables that attained statistical significance at a *p*-value of 0.05 or less were further evaluated using logistic regression analysis. Both unadjusted (UOR) and adjusted Odd’s ratios (AOR) were computed and the 95% confidence limits were constructed around the estimates.

### Ethical approval

Ethical approval was sought from the Ethical Review Committee of Ghana Health Service, Research and Development Division, Accra. Clearance to conduct the study was obtained from the District Health Directorate and the hospital management. Data abstraction was done in the conference room of the hospital during hours when it was not in use and to preclude removal of patient records from the hospital. During that time, efforts were made to restrict access to persons directly involved in the research. The trained record clerk was blinded to the research outcome to minimize subjectivity in classification. To protect the identity of the record owners, the abstraction forms did not include the patients’ names which were substituted with assigned identification numbers. The master list was stored securely in a pass worded computer, which only the authors had access to. Information from the records were not shared with other individuals outside the research team. Published reports were based on grouped data, precluding the use of individual information.

## Results

### Sociodemographic characteristics

A total of 159 cases of cervical cancer were registered from 2012 to 2016. Of these, 157 (98.74%) records were available for review, 2 records were not found. The mean age of the cases was 56.74 [± 15.11] years (range: 26 – 100 years). Among the cases, 55.19% had no formal education, while the rest attained primary education or higher (44.81%). Fifty-one percent of the cases reviewed were married. More than half (54.78%) of the cases resided in urban areas and 87.9% of them were employed. Active membership of the National Health Insurance Scheme (NHIS) was recorded among 86.84% of cases and 40% of cases lived more than 100 km from the study site (Mdn = 93.1 km; range 0.5 km – 1084 km). Patient characteristics have been summarized in Table 1.

**Table 1:** Characteristics of cervical cancer cases

Socio-demographic factors	n	Frequency	Percentage
Age (years)	157		
≤ 50		57	36.31
> 50		100	63.69
Highest level of education	154		
None		85	55.19
Primary and above		69	44.81
Marital Status	155		
Never married/single		9	5.81
Married		80	51.63
Other (Divorced/ Separated/widowed)		66	42.58
Employment Status	157		
Unemployed		19	12.10
Employed		138	87.90
Residence	157		
Rural		71	45.22
Urban		86	54.78
NHIS Status	152		
Insured		132	86.84
Not Insured		20	13.16
Distance	154		
0 – 50 km		24	15.58
> 50-100 km		68	44.16
> 100 km		62	40.26

### Characteristics associated with late stage at presentation of cervical cancer cases

The hospital based prevalence of late stage at presentation among cervical cancer cases was 65.97% (95% CI: 57.61–73.65%). Notably patients who presented late with cervical cancer and had no education comprised three-quarters of the total cases reviewed (Table 2). Of the variables tested for association, only level of education showed significant association with late stage at presentation of cervical cancer cases (χ2 = 5.7309; df = 1; *p* = 0.017).

**Table 2:** Cross tabulation of sociodemographic factors and late stage at presentation of cervical cancer cases

Socio-demographic factors	Late stage at presentation		χ^2^	*p*-value
	Yes	No		
Age (years)				
≤ 50	32(61.54)	20(38.46)		
> 50	63(68.48)	29(31.52)	0.7127	0.399
Level of education				
None	59(74.68)	20(25.32)		
Primary and above	35(55.56)	28(44.44)	5.7309	0.017*
Marital Status^§^				
Never married/single	6(75.00)	2(25.00)		
Married	49 (70.00)	21 (30.00)		
Other (Divorced/Separated/Widowed)	40 (65.50)	24 (37.50)	1.1004	0.643§
Employment Status^§^				
Unemployed	13 (81.25)	3 (18.75)		
Employed	82 (64.06)	46 (35.94)	1.8715	0.263§
NHIS Status				
Insured	80 (65.57)	42 (34.43)		
Not Insured	13 (72.22)	5 (27.78)	0.3109	0.577
Residence				
Rural	43 (68.25)	20 (31.75)		
Urban	52 (64.20)	29 (35.80)	0.2598	0.610
Distance				
≤ 50 km	14 (63.64)	8 (36.36)		
51-100 km	41 (66.13)	21 (33.87)		
> 100 km	37 (64.91)	20 (35.09)	0.0493	0.976

*P*-values were based on Pearson chi-square and Fishers exact tests for categorical variables, (%) represent column percentage, ^§^Corresponding *p*-value was estimated from Fisher’s exact test, *p-value from Pearson chi-square test, significant at 95% confidence level

The mean age at menarche was 16 years (range 7–25 years). Among the clinical characteristics, cases that presented late tended to be women who had not been screened previously for cervical cancer (*χ*^2^ = 7.2840,  *df* = 1; *p* = 0.007) and those who had menarche between the ages of 16–25 years (*χ*^2^ = 5.0264, *df* = 1; *p* = 0.025) Intermenstrual bleeding, post menopausal bleeding, history of smoking and parity were not associated with late stage at presentation of cervical cases (Table 3).

**Table 3:** Cross tabulation of clinical factors and late stage at presentation of cervical cancer cases

Clinical Variables	N (%)	Late stage presentation		χ^2^	*p*-value
		Yes	No		
Intermenstrual bleeding	150				
Yes	34 (22.67)	19 (61.29)	12 (38.71)		
No	116 (77.33)	73 (67.59)	35 (32.41)	0.4275	0.513
Post-menopausal bleeding	148				
Yes	116 (78.38)	73 (67.59)	35 (32.41)		
No	32 (21.62)	18 (62.07)	11 (37.93)	0.3127	0.576
Previous Screening	151				
Yes	23 (15.23)	9 (40.91)	13 (59.09)		
No	128 (84.77)	84 (70.59)	35 (29.41)	7.2840	0.007*
Previous Smoking§	155				
Yes	1 (0.65)	0 (0.00)	1 (100.00)		
No	154 (99.35)	93 (65.96)	48 (34.04)	1.9114	0.345^§^
Parity	147				
0–4	69 (46.40)	40 (61.54)	25 (38.46)		
≥ 5	78 (53.60)	49 (69.01)	22 (30.99)	0.8385	0.360
Age at menarche^§^	66				
7–15 years	27 (40.91)	9 (39.13)	14 (60.87)		
16–25 years	39 (59.09)	26 (68.42)	12 (31.58)	5.0264	0.025*

Percentages-in parentheses; *p-values were based on Pearson chi-square and Fishers exact test for categorical variables; (%) represent column percentage, under Late stage presentation – row percentages are presented, ^*§*^*p*-value estimated from Fisher’s exact test, *statistically significant when *p* < 0.05

Variables attaining statistical significance (level of education, age at menarche and previous screening history) were included in a regression model, adjusting for age. Sensitivity analysis was performed to assess the effect of missing values from age at menarche, *n* = 66 (see Additional file 1). In the first scenario, all the missing records were assumed to belong to women who attained menarche between ages 7–15 years. In the second scenario, all the missing records were assumed to belong to women who attained menarche between ages 16–25 years. Results showed that adjusted effect estimates for age (Scenario 1: OR1.05, 95% CI 0.47–2.33; Scenario 2: OR 0.89, 95% CI 0.39–2.03), level of education (Scenario 1: OR 0.46, 95% CI 0.22–1.10; Scenario 2: OR 0.51, 95% CI 0.23–1.12) and screening history (Scenario 1: OR 4.07, 95% CI 1.47–11.26; Scenario 2: OR 3.40, 95% CI 1.43–11.16) did not change substantially. Therefore, these variables were maintained in the final model (Table 4). Age at menarche was eliminated from the adjusted model. After adjusting for age and level of education, previously unscreened cases of cervical cancer were nearly four times more likely to present late, compared to patients who were screened earlier (OR 3.91, 95% CI:1.43–10.69) (Table 4). None of the histological characteristics was associated with late stage at presentation of cervical cancer cases (Table 5).

**Table 4:** Factors associated with late stage at presentation of cervical cancer cases

Covariate	Late stage at presentation of cervical cancer cases					
	Unadjusted effect			Adjusted effect		
	UOR	95%CI	*p*-value	AOR	95%CI	*p*-value
Age						
≤ 50 years	ref			ref		
> 50 years	1.43	0.70–2.92	0.327	1.04	0.47–2.30	0.927
Educational level						
No education	ref			ref		
Primary and above	0.45	0.22–0.92	0.029*	0.46	0.22–1.02	0.055
Previous screening						
Yes	ref			ref		
No	3.61	1.41–9.23	0.007*	3.91	1.43–10.69	0.008*

*UOR* Unadjusted Odd’s Ratio*, AOR* Adjusted Odd’s Ratio*, 95% CI* 95% confidence level, * statistical significance at 95% confidence level

**Table 5:** Cross tabulation of histological factors and late presentation with cervical cancer

Histological Factors	N (%)	Late Stage Presentation		χ2	*p*-value
		Yes	No		
Type of tumour	148				
Squamous Cell Carcinoma	116 (78.38)	70 (66.04)	36 (33.96)		
Adenocarcinoma/ Adenosquamous/ Other rare types	32 (21.62)	19 (63.33)	11 (36.67)	0.0756	0.783
Characteristics of tumour	41				
Well differentiated	11 (26.83)	7 (70.00)	3 (30.00)		
Moderately differentiated	20 (48.78)	15 (78.95)	4 (21.05)		
Poorly differentiated	8 (19.51)	4 (57.14)	3 (42.86)		
Undifferentiated	2 (4.88)	1 (50.00)	1 (50.00)	1.6706	0.500^§^

*P*-values in parentheses, (%) represent column percentage, ^*§*^*p*-value estimate from Fisher’s exact test

## Discussion

The study investigated the range of sociodemographic, clinical and histological factors associated with late stage presentation of cervical cancer cases who reported to Gynecological Oncology Care Unit at the Catholic Hospital, Battor from 2012 to 2016. The findings suggest that sociodemographic (no education) and clinical factors (absence of previous screening and age at menarche) influenced late presentation at the study site. Nearly two-thirds of patients with cervical cancer during the stated period reported with advanced stage disease, Similar results were found in studies conducted in Uganda, South India, Iran, Nepal, Tanzania [4–6, 13, 14] which make it evident that majority of women report with late stage disease, particularly in developing countries.

Other sociodemographic factors (age, marital status, educational level, NHIS, employment status, place of residence and distance) were not associated with late stage at presentation of cervical cancer cases. The influence of education was reported in similar studies conducted in Tanzania [5], Nepal [6] and Morocco [16]. This means that women who are educated will be more inclined to report early once they experience an unusual symptom. As most women with cervical cancer present at older ages, age was included to assess how it might affect other factors in the multivariable analysis. In this study, age was not a predictor of late presentation. This finding which contrasts with findings from earlier studies [8, 10, 16] agrees with a study in Uganda [4]. In terms of access to healthcare, active membership of the National Health Insurance Scheme was included in the present study. Nevertheless, being uninsured was not associated with late presentation. A potential explanation could be that National Health Insurance does not cover cost of diagnosis, but total abdominal hysterectomy as treatment for early cervical cancer. Moreover, women were dissuaded from seeking medical help related to gynecological issues based on the need to self-fund until symptoms become worse. In other studies, insurance covered the full management of cervical cancer [10–12].

**Fig. 3:**
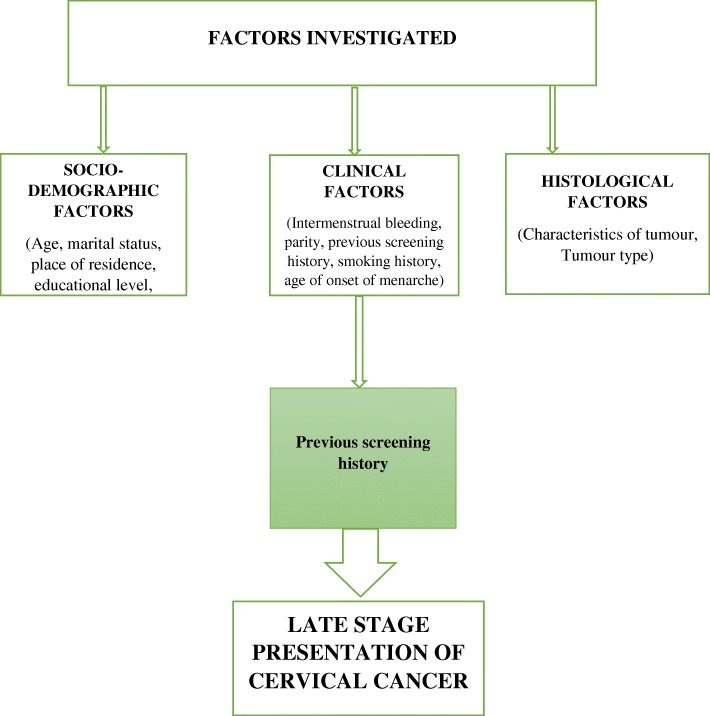
Framework explaining late presentation of cervical cancer cases at Catholic Hospital, Battor

The hospital is surrounded by rural communities, women who seek gynecological cancer care come from both rural and urban settings. Studies in the United States [15], Sudan [10] and Morocco [16] reported an association with rural urban residence, while studies in Uganda [4], and Nepal [6] did not report any association as in the present study. Being that the present study was conducted retrospectively, ascertaining average monthly income was not possible hence employment status was used as a proxy. Income generation can be used in determining the socioeconomic status of an individual as low socioeconomic status has been found to have a pronounced effect on late presentation [4, 14]. Employment status was used as a proxy measure to indicate income generation and suggests that a woman might have the capacity to care for her own health rather than relying on her partner or family relations, as in the case of unemployed women. Interestingly, this factor did not predict late stage at presentation with cervical cancer, compared to studies conducted in Uganda [4] and Iran [14]. It is expected that due to lack of money, women will not seek early care until symptoms becomes worse. Among all clinical variables (intermenstrual bleeding, previous screening history, smoking history, number of children and age at menarche) studied, only cases that did not undergo previous screening for cervical cancer and cases that reported older ages at menarche were independently associated with late presentation. A previous study in Iran [14] also reported a significant association with not having been screened previously. Women who have cervical lesions that are not detected early may progress to advanced disease without intervention. The older age at menarche was an unusual finding as it has not been reported previously in local studies or in the studies reviewed in this research. As the statistical association was not maintained in the results from the sensitivity analysis, this variable was omitted from the adjusted model. The authors have reconstructed the original conceptual framework to summarize the study results (Fig. 3).

### Limitations

It is noteworthy that this study had some limitations. Firstly, the study being a retrospective study relied solely on the medical records. No additional information could be collected as the patients were not physically present. For example; although literature reports HIV status, contraceptive use and sexually transmitted infections as relevant clinical factors associated with cervical cancer, these were not documented in the records by the attending medical personnel in the period under study. Additionally, some missing data were observed for most of the variables investigated. This particularly affected age at menarche and the histopathology results. The missing data on age at menarche can be attributed to some sociocultural factors such as lack of education about menstruation by parents and guardians due to embarrassment in addressing the subject. There is also much emphasis placed on instilling fear about getting pregnant, rather than keeping personal record of gynaecological information such as menarche. The histopathology results often did not report on the tumour sub-type and characteristics of tumour. These omissions might have resulted from either the record personnel, the attending physician or the patient if she was unable or unwilling to supply the required information. Other cases that may not have reported at the facility in the time frame were excluded, so the actual prevalence could probably be higher.

A sensitivity analysis was conducted to assess the effect of the extent of missing values on the logistic regression model. In the first scenario, it was assumed that all missing records of age at menarche belonged to women who attained menarche in the age range 7–15 years. In the second scenario, it was assumed that all the missing records of age at menarche belonged to women who attained menarche in the age range 16–25 years. In both models, the absence of previous screening remained a significant factor associated with late stage at presentation of cervical cancer cases. However, age at menarche was not always associated with late presentation of cervical cancer. Therefore, the extent of missing values for age at menarche makes it difficult to conclude definitively that age at menarche is associated with late presentation of cervical cancer.

### Future directions

In view of the findings, the following are recommended at community level:

1. The creation of an advocacy tool using a pictorial illustration to highlight study findings. This can be employed to create awareness and support cancer education at the Gynecological Oncology care unit. The tool should show the link between absence of screening and late stage at presentation. This would explain why women need to be screened early. Simple information should be included about places where women can get screened and time of availability of the service. The tool can be field tested and replicated for use during home visits made by community health workers and should target adult women. Further research will be required to assess the impact of the tool in improving screening rates in Battor, using the present study as a baseline.
2. Focus groups can be held with male and female groups to explore barriers to screening and find local solutions to address them. Engaging the support of male partners for increased uptake of preventive screening by women is a pro-active step. This is because male partners can provide funds to pay for the service and thereby influence health seeking behaviour.
3. The study findings should be disseminated. The media and local durbars offer the opportunity to increase awareness and advocate support from donor groups, civil society and private agencies to help equip additional service units so that services can have a wider reach. The provision of gift vouchers to facilitate screening and subsidize treatment costs by corporate organizations would be a meaningful addition.
4. The relationship with age at menarche can be explored further in future research if women are informed about the importance of keeping relevant information.

At the national level, the following are recommended:

1. Training of public health nurses, physician assistants and community health workers in preventive screening and supporting them with required logistics could be a way to decentralize services and make screening more accessible to women. This can be organized by Ghana Health Service in collaboration with local oncologists.
2. Consideration should be given to the inclusion of cancer screening and early treatment under the National Health Insurance Scheme. This is recommended as an adjunct measure to other recommendations as its merit lacked statistical support in the study, but it can offer potential benefits.
3. Standard guidelines for screening should be provided in consulting rooms and/or service units which should be supported with the necessary logistics.
4. Health education for in-school and out of school youth should provide information about keeping personal records of important developmental landmarks like menarche in the case of females. Physically challenged adolescent females can be assisted by teachers, guardians or a significant other person to maintain relevant health records, which should help attending physicians capture their data for analysis. Health workers can only record information that is provided by their patients voluntarily. Women will be encouraged to offer such information provided that they understand why it is relevant to keep account of it.

## Conclusion

Three factors tended to be associated with late stage presentation of cervical cancer cases at Battor, namely lack of formal education, the absence of previous screening and older age at menarche. However, only absence of previous screening was predictive of late stage presentation of cervical cancer cases. As nearly two thirds of the cases presented in advanced stages of cervical cancer, efforts to promote early cervical cancer screening should be intensified. Further studies are required to investigate a relationship with age at menarche.

## Additional file

Additional file 1: Authors’ original file for the sensitivity analysis showing two scenarios. (DOCX 18 kb)

## Abbreviations

95% CI: 95% Confidence interval
AOR: Adjusted odd’s ratio
DNA: Deoxyribonucleic acid
NHIS: National Health Insurance Scheme
UOR: Unadjusted odd’s ratio
VIA: Visual Inspection with Acetic Acid
