# Correction to: Factors associated with late presentation of cervical cancer cases at a district hospital: a retrospective study

**DOI:** 10.1186/s12889-018-6127-9

**Published:** 2018-11-07

**Authors:** Priscilla Dunyo, Kofi Effah, Emilia Asuquo Udofia

**Affiliations:** 1 Department of Population, Family and Reproductive Health, School of Public Health, University of Ghana, Legon, Accra, Ghana; 2 Obstetric and Gynecological Department/Cervical Cancer Screening and Training Center, Catholic Hospital, Battor, Ghana; 3 Department of Community Health, School of Public Health, University of Ghana, Legon, Accra, Ghana

## Correction to BMC Public Health (2018) 18:1156 DOI: 10.1186/s12889-018-6065-6

It has been highlighted, that the original article [1] contained errors in Fig. 1. On the 3rd level, the first box should read “Records with any required information = 157” and the second box should read “Records without any required information = 0”. Those were incorrectly captured as “Records with complete information = 157” and “Records without any required information = 157” respectively in the original article. This Correction article shows the correct Fig. 1.

**Fig. 1:**
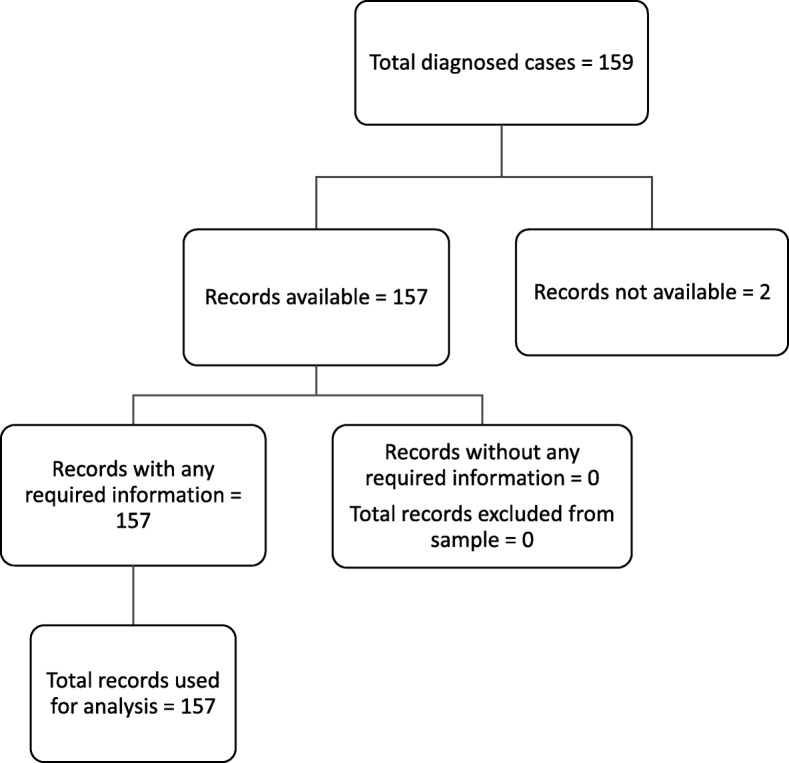
Schematic diagram showing the record selection process
