# Supplementary material for: Factors associated with late presentation of cervical cancer cases at a district hospital: a retrospective study
Source: BMC Public Health. 2018 Oct 3;18:1156. doi: 10.1186/s12889-018-6065-6 (PMC6171232; doi:10.1186/s12889-018-6065-6)
Supplement: Supplementary file 1 — Authors’ original file for the sensitivity analysis showing two scenarios. (DOCX 18 kb) [file 12889_2018_6065_MOESM1_ESM.docx]

SENSITIVITY ANALYSIS FOR AGE AT MENARCHE

**Scenario 1: Factors associated with late stage at presentation of cervical cancer cases (assuming all missing records for age at menarche = 7-15 years)**

|  | Late stage at presentation of cervical cancer cases | | | | | |
| --- | --- | --- | --- | --- | --- | --- |
|  | **Unadjusted effect** | | | **Adjusted effect** | | |
| Covariate | **UOR** | **95%CI** | **p-value** | **AOR** | **95%CI** | **p-value** |
| Age |  |  |  |  |  |  |
| ≤50 years | ref |  |  | ref |  |  |
| >50 years | 1.43 | 0.70 – 2.92 | 0.327 | 1.05 | 0.47 – 2.33 | 0.906 |
|  |  |  |  |  |  |  |
| Level of education |  |  |  |  |  |  |
| No education | ref |  |  | ref |  |  |
| Primary and above | 0.45 | 0.22 – 0.92 | **0.029*** | 0.46 | 0.22 – 1.10 | 0.572 |
|  |  |  |  |  |  |  |
| Previous screening |  |  |  |  |  |  |
| Yes | ref |  |  | ref |  |  |
| No | 3.61 | 1.41 – 9.23 | **0.007*** | 4.07 | 1.47 – 11.26 | **0.007*** |
|  |  |  |  |  |  |  |
| Age at menarche |  |  |  |  |  |  |
| 7 – 15 years | ref |  |  | ref |  |  |
| 16 – 25 years | 1.11 | 0.50 – 2.46 | 0.789 | 1.38 | 0.58 – 3.26 | 0.461 |

*UOR = Unadjusted Odd’s Ratio; AOR = Adjusted Odd’s Ratio; 95% CI = 95% confidence level*

**Scenario 2: Factors associated with late stage at presentation of cervical cancer cases (assuming all missing records for age at menarche = 16-25 years)**

|  | Late stage at presentation of cervical cancer cases | | | | | |
| --- | --- | --- | --- | --- | --- | --- |
|  | **Unadjusted effect** | | | **Adjusted effect** | | |
| Covariate | **UOR** | **95%CI** | **p-value** | **AOR** | **95%CI** | **p-value** |
| Age |  |  |  |  |  |  |
| ≤50 years | ref |  |  | ref |  |  |
| >50 years | 1.43 | 0.70 – 2.92 | 0.327 | 0.89 | 0.39 – 2.03 | 0.775 |
|  |  |  |  |  |  |  |
| Level of education |  |  |  |  |  |  |
| No education | ref |  |  | ref |  |  |
| Primary and above | 0.45 | 0.22 – 0.92 | **0.029*** | 0.51 | 0.23 – 1.12 | 0.093 |
|  |  |  |  |  |  |  |
| Previous screening |  |  |  |  |  |  |
| Yes | ref |  |  | ref |  |  |
| No | 3.61 | 1.41 – 9.23 | **0.007*** | 3.40 | 1.43 – 11.16 | **0.008*** |
|  |  |  |  |  |  |  |
| Age at menarche |  |  |  |  |  |  |
| 7 – 15 years | ref |  |  | ref |  |  |
| 16 – 25 years | 3.40 | 1.58 – 10.0 | **0.003*** | 3.73 | 1.40 – 9.97 | **0.009*** |

*UOR = Unadjusted Odd’s Ratio; AOR = Adjusted Odd’s Ratio; 95% CI = 95% confidence level*
